# Antitumor activity of a rhenium (I)-diselenoether complex in experimental models of human breast cancer

**DOI:** 10.1007/s10637-015-0265-z

**Published:** 2015-06-26

**Authors:** Philippe Collery, Ahmed Mohsen, Anthony Kermagoret, Samantha Corre, Gérard Bastian, Alain Tomas, Ming Wei, François Santoni, Nadia Guerra, Didier Desmaële, Jean d’Angelo

**Affiliations:** Société de Coordination de Recherches Thérapeutiques, Algajola, France; Faculté de Pharmacie, Université Paris-Sud, Institut Galien, UMR CNRS 8612, Chatenay-Malabry, France; Faculté de Pharmacie, Université Paris-Sud, UMR CNRS 8076 BIOCIS, Chatenay-Malabry, France; Department of Life Science, Imperial College of London, London, UK; Département de Pharmacologie, Centre Hospitalier Universitaire Pitié-Salpêtrière, Paris, France; Laboratoire de Cristallographie et RMN, Faculté de Pharmacie, UMR CNRS 8015, Université Paris Descartes, Paris, France; Laboratoire Cellvax, Ecole Vétérinaire Nationale d’Alfort, Maisons Alfort, France; Laboratoire de l’Office d’Equipement Hydraulique de Corse, Bastia, France

**Keywords:** Rhenium, Selenium, Breast cancer, MDA-MB231 cell line, Bioluminescence

## Abstract

Rhenium (I)-diselenother (Re-diselenoether) is a water soluble metal-based compound, combining one atom of rhenium and two atoms of selenium. This compound has been reported to exhibit marked activities against several solid tumor cell lines. We now disclose an improved synthesis of this complex. The Re-diselenoether showed a potent inhibitory effect on MDA-MB231 cell division in vitro, which lasted when the complex was no longer present in the culture. Re-diselenoether induced a remarkable reduction of the volume of the primitive breast tumors and of the pulmonary metastases without clinical signs of toxicity, in mice-bearing a MDA-MB231 Luc+ tumor, orthotopically transplanted, after a daily oral administration at the dose of 10 mg/kg/d. Interestingly, an antagonism was observed when cisplatin was administered as a single i.p. injection 1 week after the end of the Re-diselenoether administration. In an effort to gain insight of the mechanisms of action of Re-diselenoether complex, interaction with 9-methylguanine as a nucleic acid base model was studied. We have shown that Re-diselenoether gave both mono- and bis-guanine Re adducts, the species assumed to be responsible for the DNA intrastrand lesions.

**Electronic supplementary material:**

The online version of this article (doi:10.1007/s10637-015-0265-z) contains supplementary material, which is available to authorized users.

## Introduction

**Fig. 1:**
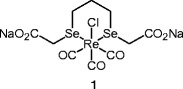
Chemical structure of complex **1**

Metal-based drugs have received increasing attention in recent years. The use of metals is indeed very attractive, as they offer unique spectrum of reactivity through ligand exchange and redox processes that is not available in the more common organic-based drugs. The discovery of the anticancer properties of cisplatin during the 1960s spurred the quest for alternative anticancer drugs with less side-effects. Beside platinum analogues including platinum (II) and (IV) derivatives, other metals have been recently explored, such as gallium, ruthenium, iron, gold, titanium or palladium [1]. In this context, rhenium-based drugs appeared as promising candidates for clinical development. Over the past years a growing number of studies have revealed the potential of Re organometallic complexes as anti-cancer agents. A recent review has been published with a particular emphasis on the cellular uptake and the localization of the currently known Re organometallic complexes as well as their potential mechanism of action [2]. Among Re organometallic complexes, several Re carbonyl complexes have been found to display cytotoxicity against breast cancer cell lines. For example, a Re(tricarbonyl)pentylcarbonato compound able to fight triple node negative human breast cancer cell lines has been described [3]. Nevertheless, despite the design of very efficient potent anti-cancer agents, very few in vivo studies have been conducted on *cold* Re organometallic complexes. On the other hand, it is noteworthy that some Se-based drugs have demonstrated a selective cytotoxicity against cancerous cells [4–6]. The tumor-specific cytotoxic effects of Se, with special emphasis on cascades of cellular events induced by pharmacologically active Se compounds have been recently reviewed [7]. It appears that certain redox-activated Se compounds induce complex cascades of pro-death signaling at pharmacological concentrations with superior tumor specificity, and that the target molecules are often implicated in drug resistance. With the aim to combine the antiproliferative properties of Re with the unique apoptotic modulator properties of Se we have recently designed the rhenium(I)-diselenoether complex **1** in which a central Re atom is coordinated with two Se atoms (Fig. 1). Complex **1** was shown to exhibit remarkable cytotoxicity against MCF-7 breast cancer cell lines [8]. The uptake and efflux of Re in malignant cells exposed to complex **1** have been reported, together with evidence of the incorporation of Re into the nucleus. Furthermore, tissue distribution of Re and Se after oral administration of **1** to mice have been reported [9].

The purpose of the present paper was to report the activity of Re-diselenother complex **1** (Fig. 1) in experimental models of human breast tumor toward highly metastatic MDA-MB231 cancer cells in culture, and in MDA-MB231 Luc+ tumors transplanted in mice. The interaction of **1** with 9-methylguanine is also described, providing evidence that interaction of **1** with DNA might be involved in the mechanism of action of **1** at the molecular level.

## Material and methods

### Chemical protocols

**Scheme 1:**
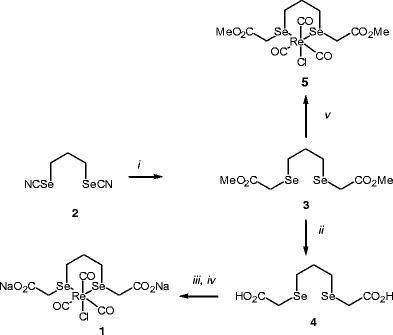
Synthesis of **1** with optimized approach to key-intermediate **4**. Reagents and conditions, *i*: BrCH_2_CO_2_Me, NaBH_4_, EtOH, 16 h, 20 °C (82 %); *ii*: LiOH.H_2_O, THF, MeOH, 16 h, 20 °C (85 %); *iii*: ReCl(CO)_5_, THF, reflux, 16 h (72 %); *iv*: 2.0 equiv. NaHCO_3_, MeOH,H_2_O, 0 °C (90 %); *v:* ReCl(CO)_5_, THF, reflux, 16 h (68 %)

Although the published synthesis of complex **1** has proved rather efficient, the elaboration of a key intermediate (compound **4** in scheme 1) was somewhat problematic, since suffering from a vexing lack of reproducibility. For that reason, we have recently developed an alternative approach to key-compound **4**, which has proved perfectly reproducible. This new protocol involved the alkylation of the disodium salt of propane-diselenocyanate **2** with bromoacetic acid methyl ester, giving diester **3**, which was next saponified with lithium hydroxide into diacid **4**. Complexation of ReCl(CO)_3_ by diselenoether **4**, followed by sodium bicarbonate treatment provided complex **1**, as previously reported [8]. Likewise, to study the possible interactions of the complex with DNA bases without competitive attack of the carboxylate appendages on the Re atom, the corresponding dimethyl ester complex **5** was prepared by condensation of diselenoester **3** with ReCl(CO)_3_ in 68 % yield_._

#### Alternative synthesis of key intermediate

Preparation of (3-carboxymethylselanyl-propylselanyl)-acetic acid dimethyl ester (compound **3**): To a solution of 1,3-bis-selenocyanato-propane **2** (1.0 g, 3.96 mmol) in absolute ethanol (20 mL) was added bromoacetic acid methyl ester (1.23 g, 8.0 mmol). The mixture was stirred under nitrogen until complete dissolution. Sodium borohydride (303 mg, 8.0 mmol) was then added in one portion. The reaction mixture was stirred at room temperature for 16 h. The white precipitate was filtered of on a sintered glass funnel and the paint yellow filtrate was concentrated under reduced pressure to leave **3** as a pale yellow oil (1.12 g, 82 %); ^1^H NMR (CDCl_3_): δ 3.72 (s, 6H, OC*H*_3_), 3.17 (s, 4H, C*H*_2_CO_2_Me), 2.82 (t, *J* = 7.2 Hz, 4H, SeC*H*_2_CH_2_C*H*_2_Se), 2.0 (quint, *J* = 7.2 Hz, 2H, SeCH_2_C*H*_2_CH_2_Se).

#### Preparation of (3-carboxymethylselanyl-propylselanyl)-acetic acid (compound **4**)

To a solution of compound **3** (692 mg, 2.0 mmol) in THF (3 mL) and methanol (1 mL) was added a solution of LiOH.H_2_O (336 mg, 8.0 mmol) in 2 mL of water. The reaction mixture was stirred at room temperature for 16 h and the solvents were removed under reduced pressure. 3 N HCl was added until pH = 1, and the mixture was extracted with ethyl acetate (3 × 15 mL). The combined organic layers were dried over MgSO_4_ and concentrated under reduced pressure. The oily material was taken into a small amount of CH_2_Cl_2_ and precipitate with petroleum ether. The solid was filtered, washed with diethyl ether, and dried under vacuum to give 540 mg (85 % yield) of compound **4**, which was unequivocally identified by comparison with an authentic sample.

#### Synthesis of dimethyl ester complex **5**

Preparation of rhenate (tricarbonylchloro[2, 2′-[1, 3-propanediylbis(carbomethoxymethylseleno-)]]) (**5**): A mixture of diselenoester **3** (216 mg, 0.62 mmol) and ReCl(CO)_3_ in THF (20 mL) was heated at 60 °C for 16 h. The mixture was cooled to room temperature and concentrated under reduced pressure to leave crude **5**. Purification by chromatography over silica gel gave **5** as a colorless oil (278 mg, 68 %); ^1^H NMR (C_6_D_6_): The presence of stereoisomers induced splitting of most signals δ 3.61 (dd, *J* = 14.1, 4.5 Hz, 1H), 3.42 (dd, *J* = 14.1, 6.9 Hz, 1H), 3.35–3.24 (m, 6H), 3.16–3.06 (m, 0.5H), 2.99 (d, *J* = 13.8 Hz, 1H), 2.86 (m, 0.5H), 2.55–2.28 (m, 2 H), 1.5–1.2 (m, 4H).

#### Interaction of rhenium diseleno-ester **5** with 9-methylguanine

To a solution of complex **5** (139 mg, 0.21 mmol) in methanol (4 mL) was added dropwise a solution of AgBF_4_ (42 mg, 0.21 mmol) in methanol (1 mL). A sticky precipitate formed immediately. The reaction mixture was stirred at 15 °C for 16 h and filtered. The filtrate was added to a solution of 9-methylguanine (35 mg, 0.21 mmol) in 1:1 water: methanol mixture (24 mL). The reaction mixture was stirred for 3 d at 15 °C and concentrated under reduced pressure (1 mm Hg) at 20 °C. The obtained solid was washed with methylene chloride to remove any trace of free ligand and dried. Mass spectra analysis ESI (+) showed four components: *m/z* = 782.0 [C_18_H_23_N_5_O_8_ReSe_2_]^+^; 616.9 [C_12_H_16_O_7_ReSe_2_]^+^; 601.1 [C_15_H_14_N_10_O_5_Re]^+^; 435.9 [C_9_H_7_N_5_O_4_Re]^+ .^ Ions *m/z* = 782.0 and *m/z* = 616.9 showed the characteristic isotopic pattern of the ReSe_2_ fragment, whereas ions *m/z* = 601.1 and *m/z* = 435.9 ions displayed a more simple profile corresponding to a rhenium complex devoid of the diselenoether ligand.

### Morphological and inhibitory effects on MDA-MB231 breast cancer cells

#### Cell lines

MDA-MB-231 (Passage No. 13) breast cancer cell lines were kindly provided by Dr. S. Fraser and Pr. M. Djamgoz at Imperial College, London. Cells were grown as adherent monolayers in Dulbecco’s Modified Eagle Medium (Sigma), supplemented with 5 % Fetal Bovine Serum and phenol red. Cultures were maintained at 37 °C with a humidified atmosphere containing 5 % CO_2_ and were passaged using 0.25 % trypsin in DPBS (PAA) when they reached 80 % confluency. Cell number was established via haemocytometer count after dead cell exclusion using trypan blue.

#### In vitro toxicity

MDA-MB231 cells were seeded on 48-well plates at 5 × 10^4^ cells/well, allowed forming an adherent monolayer overnight and then exposed to the indicated concentrations of Re-diselenoether complex for 48 h. Cells were then washed and incubated with Re-diselenoether-free medium for a further 48 h. The effects of Re on cell viability was determined at the indicated time using haemocytometer count of live cells under light microscopy and via flow cytometry. *Proliferation*. Prior to Re treatment, MDA-MB231 cells at the concentration of 1 × 10^6^ /mL in PBS were labeled with 0.5 M of violet dye (CellTrace violet Invitrogen) for 20 min at 37 °C. The intensity of fluorescence of the violet dye was acquired on an LSRFortessa flow cytometer (BD) and analyzed with FlowJo version 9.3.1 (TreeStar, Ashland, OR, USA). Dose responses over time were analysed using GraphPad Prism software version 5.03 (GraphPad Software Inc.).

### Animal study design: oral administration of Re-diselenoether complex

This study was performed in Cellvax laboratory. In this study, one of the objectives was to look for a synergism between cisplatin and Re (I)-diselenoether [10]. Hormone-independant breast cancer MDA-MB231 cells (origin: ATCC#HTB-26™), transfected with the luciferase gene (Luc+) were orthotopically implanted into the mammary gland (fat pad) in athymic *nu/nu* nude mice (Charles River, France). With a cell viability of about 97 %, 1.0 × 10^6^ cells per mouse were injected in a volume of 50 μl/mouse. The animals were 5 to 6-week-old female, of about 20 g each, and specific and opportunistic pathogen-free. They were acclimatized for at least 7 days before the initiation of the designed study. A total of 30 mice were used for this study. Animals were housed in individual polyethylene cages, in a climate and light-controlled environment. All animals were kept under environmentally controlled housing conditions: lights on between 7:00 AM and 7:00 PM; temperature inside of the animal facility strictly maintained at 21 + 1 °C; relative humidity of 70 % throughout the entire study period, and maintained in accordance with Cellvax approved standard operation procedures (SOP) and with local Ethical Committee approval. Animals were fed with commercially available rodent food (Safe, Les Tremblats, Augy, France). Water (sterilized water) was available ad libitum.

Animals were numbered and given a unique animal identification ear notch mark. ***Ethical manager*.** A Ph.D. and Veterinary Doctor at Cellvax company assumed the function of ‘Ethical Manager’ within this project. ***Experimental groups*:** Three groups of 10 mice each for a total of 30 mice were treated. Measurable mammary tumors were observed in 18 mice at day 9 after the inoculation of the tumor cells, while no mammary tumors were observed in 12 mice. Groups were then homogeneized to have 6 mice with a measurable tumor in each group. Group 1: Cisplatin (CDDP) group: mice were treated with CDDP as a single intraperitoneal (IP) injection at a dose of 6 mg/kg on day 41 after the inoculation of the tumor cells; Group 2: Re (I) - diselenoether complex group (Re drug group): mice were daily orally treated with Re - diselenoether complex at the dose of 10 mg/kg/24 h for 4 weeks, from day 9 to day 36 after the inoculation of the tumor cells; Group 3: Re (I) - diselenoether complex and CDDP group (combined drug group): mice were daily orally treated with Re-diselenoether complex at the dose of 10 mg/kg/24 h for 4 weeks, from day 9 to day 36 after the inoculation of the tumor cells (as in group 2) and then with CDDP as a single intraperitoneal (IP) injection at a dose of 6 mg/kg on day 41 (as in group 1).

#### Oral administration of the Re compounds

The Re treatments were started on day 9 after the inoculation of the tumor cells. They were orally administered in the food instead of gavage, as it is a less stressful alternative to oral gavage [11]. Transwean was used to prepare capsules in which the Re (I)-diselenoether was incorporated. The capsules were prepared the day before treatment by mixing 1 g of transwean powder (feed rodent form of powder mixed with water forms a sort of jelly) and 1 mL of water. The mixture was then placed in the wells of a 24-well plate and stored at 4 °C. The next day, the capsules were removed from the mold with a spatula and then cut into small pieces. Re drug at a dose of 10 mg/kg was diluted in a volume of 50 μl and introduced into one of the pieces of “capsule” with a syringe, then that piece was placed in the mouse cage. Once the capsule containing the treatments were consumed normal food pellets were put into the cage until evening. This mode of administration is simple and effective. The treatments were completely consumed with no risk of overdose. ***Toxicity evaluation.*** Determination of body weight was performed twice a week for each mouse. ***Anti-tumor effect*.** The tumor growth was measured (tumor length, width and volume) twice a week by using an external caliper. The mean tumor volumes [MTV; MTV + (SD); MTV + (SEM)] were estimated. The tumor growth data was recorded for each individually identified mouse. Tumor volume was calculated by using the following formula: *V = length x width*^*2*^*/2.* An imaging by bioluminescence was performed in 2 mice of each group on days 44, 51 and 58 after the inoculation of the tumor cells. The mice were selected to have comparable tumors on day 44.

#### Statistics

Statistically evaluation of the antitumor effect was assessed by ANOVA test (One way Anova on the ranks).

## Results and discussion

### Design and synthesis of Re-diselenoether **1**

Critical to the antitumor activity of pseudo-symmetric complex **1** was the presence in its framework of a central inorganic core, in which a heavy metal atom (Re) is coordinated with two semi-metal atoms (Se). Although the canonical representation of **1** displayed high molecular symmetry, examination of the ^1^H NMR spectrum clearly indicated the slow inversion around the two selenium atoms and hence the slow chair-chair interconversion of the six-membered metallacycle abolished the apparent symmetry [8]. The chemical/biological considerations which have governed the design of this three-metal core scaffold were disclosed hereafter. A major interest of Re is related to its very low mammalian toxicity; it has thus been evoked that Re is “one of the least toxic of the metallic elements”. This low toxicity, quite surprising for a heavy metal, can be tentatively interpreted on the basis of its seven degrees of oxidation state (1 to 7) that could authorize subsequent oxidative detoxification processes. Only few data exist on the metabolism of Re compounds. However, a study of the metabolism of [^188^Re(CO)_3_(carboxycyclopentadienyl)] in mice revealed the high plasma stability of this Re compound [12]. This study also suggested that the organometallic core of this complex remained unchanged under biological environment. In full agreement with this assertion, the Re compound was essentially excreted as glycine conjugate via the renal route, without further metabolism.

Regarding the presence of two Se atoms in complex **1**, it should be mentioned that, fueled by decades of animal studies, Se could significantly reduce the incidence of cancer. This topic is now an area of intense worldwide study [13]. Nevertheless, although inorganic Se has been shown to inhibit carcinogenesis, there is a concern about toxicity, since chronic feeding of inorganic Se (e.g., selenites or selenates) at levels of> 5 ppm is toxic in rodents. However, on the basis of the pioneering work of El-Bayoumy et al. [14–16] and Sanmartin et al. [17–19], a series of synthetic Se compounds have been elaborated, in which the Se atom is connected to two carbon atoms, as in complex **1**. These compounds have proved notably more potent and much less toxic than the inorganic counterparts. In contrast to Re compounds, the metabolism of Se compounds in mice is well-documented. It was found that Se compounds which included the RCH_2_SeCH_2_R pattern in their structure, such as complex **1**, were first cleaved via the trans-selenation pathway into RCH_2_SeH metabolite that, in turn was converted into H_2_Se through the β-lyase dealkylation reaction. Both H_2_Se and CH_3_SeH are thought to be pivotal metabolites in Se-mediated cancer chemoprevention [7]. A last comment should be made on the design of **1**. Since the advantage of all ionic compounds over neutral species is their improved solubility in water, which markedly facilitates their application in biological systems, precursor **2** was ornamented at the Se-levels with two acetic acid moieties [**2**→**4**]. At the last step of the synthesis the two carboxylic acid functions were ultimately converted into water-soluble disodium salt **1** (Scheme 1).

The new procedure of synthesis of compound **1** was simple, reproducible, giving a stable product easily authenticated through its IR spectrum. The presence of a d^6^*fac*-[Re(CO)_3_]^+^ moiety in complex **1** could explain its high chemical stability. This complex is amphiphilic, soluble in water, and then easy to administer. It also possesses lipophilic properties that allow a facile diffusion across cell membranes and a good biodistribution.

### Interaction of Re-diselenoether complex with 9-methylguanine

**Fig. 2:**
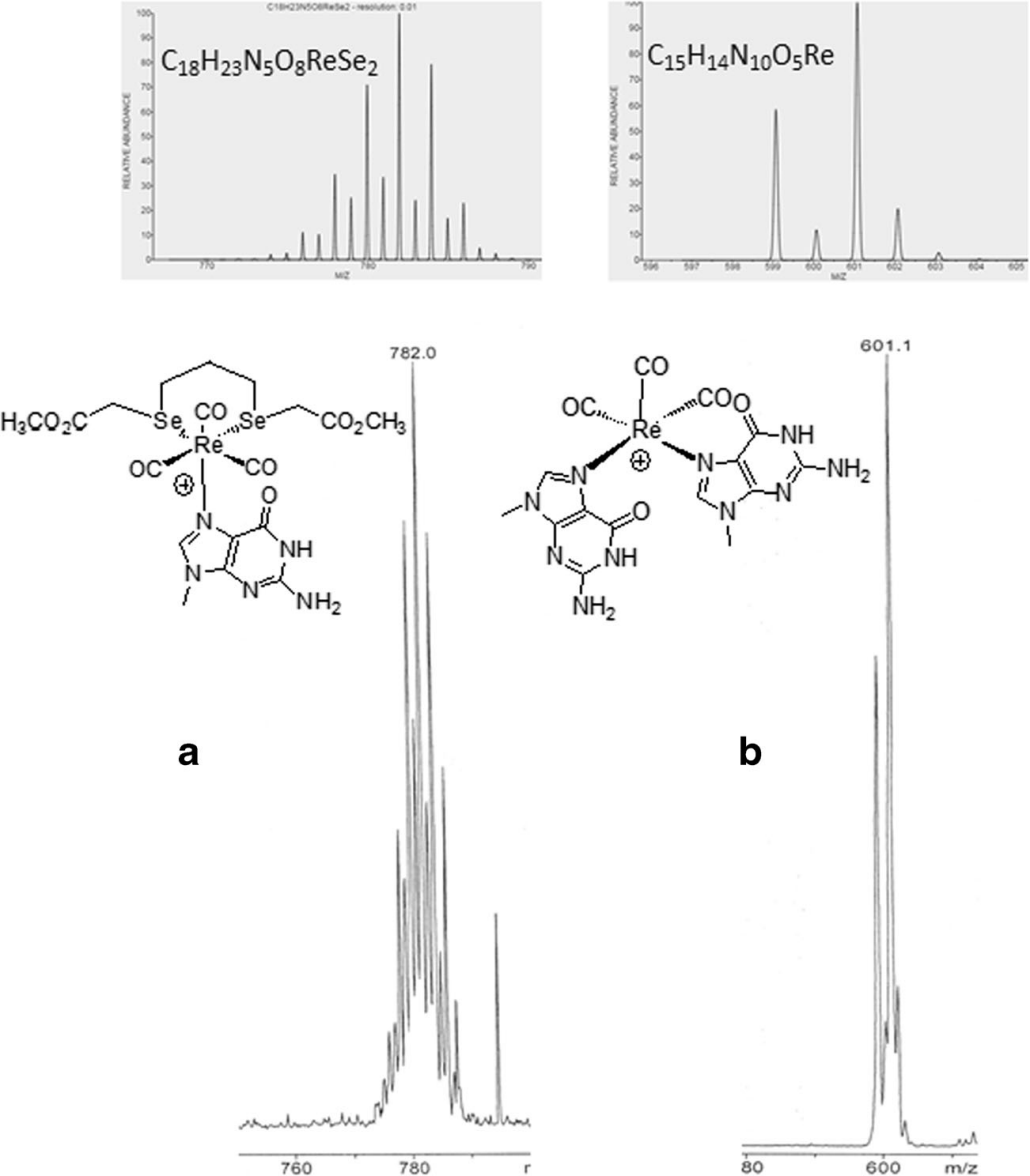
Observed (bottom) and predicted (*top*) isotopic patterns of 9-methylguanine mono-adduct ion [Re(CO)_3_(C_9_H_16_Se_2_O_4_).9-MeG]^+^ (**a**) and bis-adduct ion [Re(CO)_3_(9-MeG)_2_(H_2_O)]^+^ (**b**)

Extensive studies with many [Re(CO)_3_] complexes indicate that their cytotoxicity is due to the formation of 1,2-intrastrand adducts e.g., between the N-7 atom of two adjacent guanine residues in DNA, in a fashion similar to cisplatin. Likewise, Re accumulation in cell nucleus treated with Re-diselenoether **1** suggested a possible interaction with nucleic acids [9]. To probe such binding with complex **1**, we have investigated the reaction of **5** with 9-methylguanine (9-MeG) as simple surrogate of the guanine base in DNA. Dimethyl ester **5** was used as surrogate of complex **1**, since the presence of two sodium carboxylates in latter compound was clearly incompatible with the coupling conditions. Indeed, Alberto and Zobi had previously reported that the [Re(CO)_3_]^+^ cation bound to 9-MeG to give mono or bis-adducts [20, 21]. Thus, reaction of **5** with silver fluoroborate gave the corresponding cation which was further reacted with 1 equiv. of 9-MeG in methanol/water mixture. Analysis of the obtained mixture by mass spectrometry indicated that mono-adduct [Re(CO)_3_(C_9_H_16_Se_2_O_4_).9-MeG]^+^ BF_4_^−^ (A, Fig. 2) was indeed formed as a minor component (10–15 %). Interestingly, the major product turned out to be the bis-adduct [Re(CO)_3_(9-MeG)_2_(H_2_O)]^+^ BF_4_^−^ (B), previously observed by Alberto and Zobi. The isotopic profiles of both ions are in full agreement with the predicted patterns for the proposed molecular formulas (Fig. 2). In addition, the infrared spectrum of B revealed characteristic CO vibrations at 2027, 1915 and 1895 cm^−1^ previously reported for this 9-MeG bis-adduct [20]. These observations clearly indicated that the bis-selenoether ligand could be easily displaced by nucleic acid bases to provide guanine bis-adducts, suggesting that the Re-diselenoether complex **1** would be able to form intrastrand lesions. We may hypothesized that in biological medium, following initial aquation, the intermediate Re cation is able to react with nucleic acid bases in nucleus to give mono-adduct (Ion A, Fig. 2; Ion A, Supplementary Fig. 1). The constraint nature of the latter probably facilitated the exchange of the weakly chelating diselenoether ligand by water to give a very reactive ion (Ion D, Supplementary Fig. 1). Finally, a second nucleic acid base addition can easily take place to give 1,2-intrastrand adducts (Ion B, Fig. 2; Ion B, Supplementary Figure 1, whereas the liberated seleno ligand would probably diffuse throughout the cell.

### Antitumor effect in vitro of the Re-diselenoether complex **1**

It was earlier shown that MCF-7 breast malignant cells were more sensitive to the Re-diselenoether complex **1** than A 549 lung cancer cells and HeLa cervix carcinoma cells [9]. We therefore investigated malignant cells derived from a human breast carcinoma, the hormone-independent MDA-BB231 cells for the morphological and inhibitory effects of the Re-diselenoether complex.

**Fig 3:**
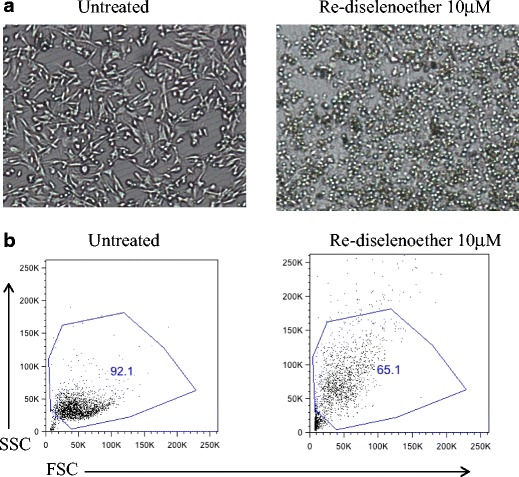
**a** Light microscopy images (5x objective) and (**b**) Flow cytometry dot plots comparing MDA-MB231 cells that have been exposed or not to 10 μM of the Re-diselenoether complex for 48 h

In the presence of a low concentration of Re (I)-diselenoether complex (10 μM), modifications in cell shape and morphology were clearly visible (Fig. 3a) when compared to untreated cells. These cellular alterations corresponded to a reduction in size and a loss of adherence indicating that treated cells were no longer proliferating and possibly included apoptotic cells. Further analysis was performed using Flow cytometry where Forward Scatter (FSC) and Side Scatter (SSC) parameters correspond to measurements of cell size (FSC) and granularity (SSC) (Fig. 3b). Cellular and nuclear debris generated by dead cells were characterized by low FSC/SSC values (<30 K) and excluded from the live gate. Upon 48 h exposure to Re, only 65.1 % of Re-treated cells were identified as alive compared to 92.1 % in the untreated condition. This heterogeneous population included cells of low FSC, indicative of non-proliferative cells, and cells of high SSC, indicative of granular apoptotic cells, which supported the microscopic observations (Fig. 3a).

**Fig. 4:**
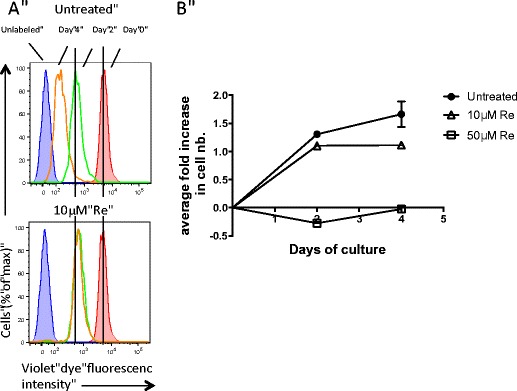
**a** Flow histograms of MDA-MB231 cells untreated or treated with 10 μM Re and analyzed for violet fluorescence intensity at the indicated time points. Plots show histograms of unlabeled control cells (*shaded blue*), cells labeled with violet dye at d0 prior culture (*shaded red*) and cells with decreasing amount of violet dye at d2 (*green*) and d4 (*orange*) due to a dilution of the violet dye upon cellular division. Data are representatives of 3 independent experiments. **b** Average fold increase in cell number over 4 days of culture in the presence of 0, 10 or 50 μM of Re complex. Data represents the mean ± SEM values pooled from 2 independent experiments

One of the hallmarks of cancer cells is their dysregulated proliferation. To further evaluate the effect of the Re complex on tumor cell ability to proliferate, MDA-MB231 cells were cultured for 48 h in the presence of 10 μM of Re complex, and for an additional 48 h in Re-free medium. A cell trace violet dye was used to label the cells prior exposure to Re and fluorescence intensity was analysed by flow cytometry (Fig. 4a). Labeled cells at d0 showing maximal fluorescence intensity (shaded red histogram) and unlabeled cells (shaded blue histogram) were used as controls. As expected, untreated MDA-MB231 cells showed cell divisions characterized by a reduction in violet dye fluorescence intensity at d2 and an even greater reduction at day 4 (upper panel). Interestingly, MDA-MB231 cells treated for 48 h with Re showed a reduction in fluorescence intensity at d2, yet to a lesser extent than untreated cells (lower panel). Most importantly, there was no further reduction at d4 indicating no further proliferation. These data show that the Re complex had a negative impact on cell division within the 48 h of culture; this inhibition was not reversed by the absence of Re in the culture beyond 48 h.

Cell division was also quantified by standard cell counts at d2 and d4 of culture with 10 and 50 μM of Re complex (Fig. 4b). Exposure to a concentration of 50 μM of Re complex had a striking effect on cell proliferation showing a total inhibition of cell division as early as 48 h which lasted after removal of the Re complex. In line with the flow cytometry experiment (Fig. 4a), the lower concentration of 10 μM of Re-drug was effective to slow down cell proliferation within 48 h of culture and prevented the cells to proliferate further beyond that time point. Altogether, these data show that the Re-diselenoether complex is a potent inhibitor of tumor cell division at low concentration and that this effect is sustained even when the complex is no longer present in the culture.

### Potential targets of the Re-diselenoether complex **1**

It has been reported that Re-based drugs could target more specifically the malignant cells than the healthy cells [2, 22]. On the other hand, it is noteworthy that some Se-based drugs have demonstrated a selective cytotoxicity against cancerous cells [4–6]. The tumor-specific cytotoxic effects and the cascades of cellular events induced by the major groups of pharmacologically active Se compounds have been reviewed [7]. It is clear that certain redox-active Se compounds induce complex cascades of pro-death signaling at pharmacological concentrations with superior tumor specificity, and that the target molecules are often implicated in drug resistance. This review also emphasized on the chemotherapeutic applications of Se with multi-target attacks on tumor cells, and moreover on the great pharmacological potential of Se for the treatment of resistant cancers.

The Re capture was previously studied in the nucleus of three human breast cancer cells [9]: MCF-7s (sensitive cells), MCF-7R (resistant cells) and MCF-7 MDR (multidrug resistant cells) were exposed to the Re-diselenoether drug at the dose of 400 μM for 48 h (uptake), followed by a post-exposure period of 48 h (efflux). The next intra-nuclear Re concentrations (μM/10^6^ cells) were recorded: MCF-7 s: 0.08 (uptake), 0.25 (efflux), MCF-7R: 0.25 (uptake), 0.12 (efflux) and MCF-7 MDR: 0.15 (uptake), 0.09 (efflux). Regarding the uptake of Re, the concentration in the nucleus was less important in the MCF-7s sensitive cells than in the other cell types. However, in MCF-7R and in MCF-7 MDR, which are MCF-7 cells with an acquired resistance to cytotoxic agents, the nucleus concentration in Re notably decreased after the post-exposure period, indicating an efflux of Re out of the nucleus. This observation is of critical importance regarding the therapeutic protocol. Indeed, with the aim of overcoming the dramatic consequences of a Re efflux in these nuclei, it appeared necessary to maintain a continuous exposure of the malignant cells to the drug; this may be achieved through a daily oral administration.

### Antitumor effect of the rhenium-diselenoether **1** in MDA-MB231 tumor-bearing mice

**Fig. 5:**
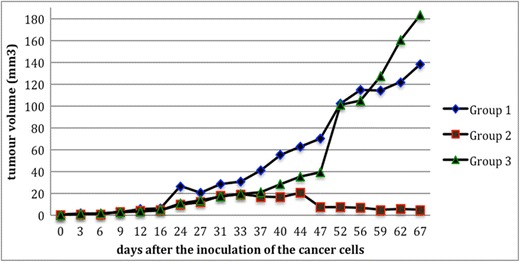
Volume of the tumors after the inoculation of the cancer cells. Group 1: treatment by cisplatin (control); group 2: treatment with Re-diselenoether complex; group 3: treatment by Re-diselenoether complex + cisplatin

It is known that liposomal rhenium cluster compounds potentiate a platinum-based chemotherapy [22–25]. For that reason, we decided to investigate a potential synergism between cisplatin and the Re-diselenoether complex in our experimental model with transplanted MDA-MB231 Luc+ human breast tumor in mice. Three groups were thus compared: Group 1, treatment by cisplatin (control); group 2, treatment with Re-diselenoether complex; group 3, treatment by Re-diselenoether complex + cisplatin. Re-diselenoether complex showed remarkable antitumor effects versus cisplatin-based chemotherapy in mouse model of breast cancer. The volume of the primitive tumor was remarkably reduced in mice treated with Re-diselenoether complex versus those treated by cisplatin, taken as a control group (p = 0.0006). Results are depicted in Fig. 5 and in Table 1 (Supplementary Table 1).

A first divergence between the three curves was observed on d16, that is to say, 2 weeks after tumor grafting. In group 1 (mice treated with a single administration of cisplatin on d41), a regular tumor volume increase was recorded at the d16-d67 interval, with a final volume reaching up to 140 mm^3^. Regarding group 2 (mice treated daily with the Re drug from d9 to d36), a plateau was observed at the d16-d44 interval, with a tumor volume not exceeding 20 mm^3^, followed by a complete regression of the tumors at the d47-67 interval. In group 3 (mice treated with the Re drug according to group 2 protocol, combined with a single administration of cisplatin on d41), the tumor volumes approximately matched those of group 2 until cisplatin administration, followed by a rapid increase until d67, with a final volume reaching up to 180 mm^3^. These observations deserve the following comments. The absence of any significant inflexion in the profile of curve 1 after cisplatin administration clearly reflected a lack of antitumor activity of this Pt-drug toward MDA-MB231 tumor-bearing mice with this schedule of treatment. By contrast, the profile of curve 2 revealed a nearly immediate antitumor effect of the Re drug, and a complete cure of the tumors after a one-month drug exposure, followed by a post-exposure period of 2 to 3 weeks. Examination of the profile of curve 3 is of peculiar interest regarding the mechanistic aspect of Pt-drugs and Re-drugs. Indeed, to our great surprise, a deleterious effect was observed when Re-drug **1** was co-administered with cisplatin, namely a dramatic collapse of the antitumor activity. This phenomenon can be interpreted on the basis of the binding modes of these metal-based drugs to DNA nucleotides which suggest that a Pt-drug could irreversibly displace a Re-drug from a pre-existing DNA-adduct. This assertion was reinforced through the consideration that both Re-drugs and Pt-drugs target the same recognition sites in DNA bases, exemplified by the N7 center of guanine.

#### Bioluminescence imaging in mice

**Fig. 6:**
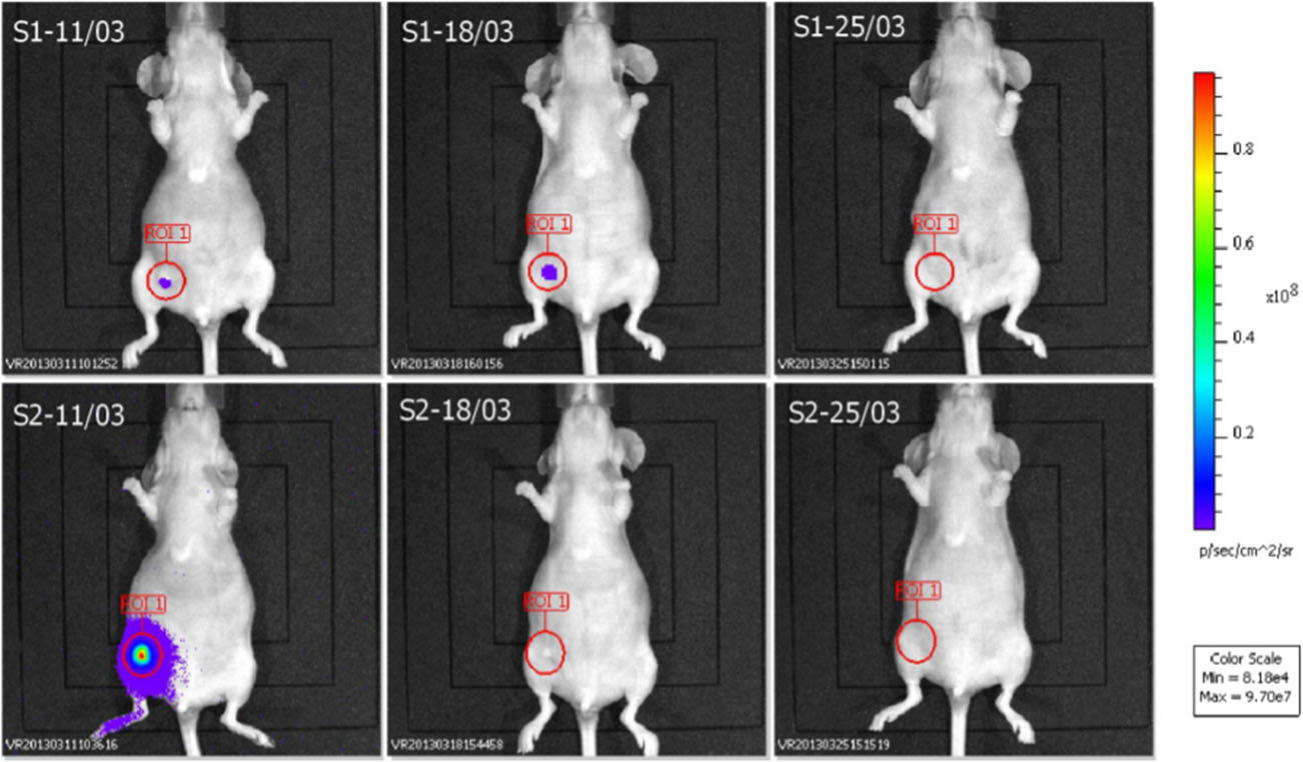
Imaging by bioluminescence in two mice (S1 and S2) treated by Re-diselenoether complex (from day 9 to day 36), on days 44 (11/03), 51 (18/03) and 58 (25/03)

The imaging by bioluminescence (Prof. Valérie Rouffiac, Institut Gustave Roussy, France) illustrated the effects of Re (I)-diselenoether complex on the tumor activity (Fig. 6). In the group of the two mice (S1 and S2) treated with the Re drug, the tumor was visible on the first imaging on day 44 after the inoculation of the cancer cells (S1-S2, 11/03 images). On day 51 (18/03), the tumor has disappeared in mouse S2. On day 58 (25/03), there was no detectable tumor in the two mice, indicating a complete regression of the tumor activity, an effect sustained a long time after the interruption of the treatment with the Re-diselenoether complex (end of the treatment on day 36 after the inoculation of the cancer cells).

#### Incidence of Re-diselenoether complex on pulmonary metastases

**Fig. 7:**
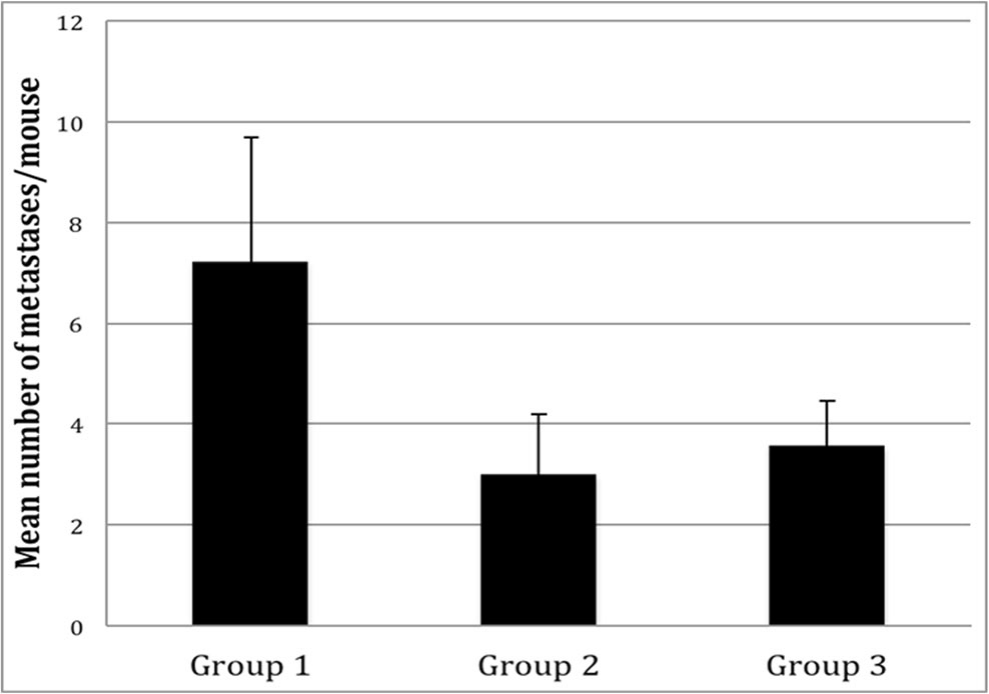
Pulmonary metastases. Group 1: mice treated by cisplatin, group 2: mice treated by Re (I) diselenoether complex, group 3: mice treated by Re (I)-diselenoether complex + cisplatin

The pulmonary metastases could be evaluated in 9 mice of each group (one mouse in each group died before day 42 and the number of pulmonary metastases was not measured). Seven suffering mice were sacrificed on day 65 (two from group 1, one from group 2 and one from group 3: they all had pulmonary metastases). All other mice were sacrificed on day 67, corresponding to the end of the study. Finally, the presence of pulmonary metastases was noted in 9/9 mice in group 1; 5/9 in group 2 and 7/9 in group 3, with a mean number of metastases of 7.22 ± 2.47 in group 1; 3 ± 1.2 in group 2 and 3.57 ± 0.90 in group 3, as represented in Fig. 7. The number of metastases was significantly greater in group 1 versus group 2 (*p* < 0.05) and in group 1 versus group 3 (*p* < 0.05), but there was no significant difference in group 2 versus group 3.

#### Evaluation of the toxicity of Re-diselenoether complex

**Fig. 8:**
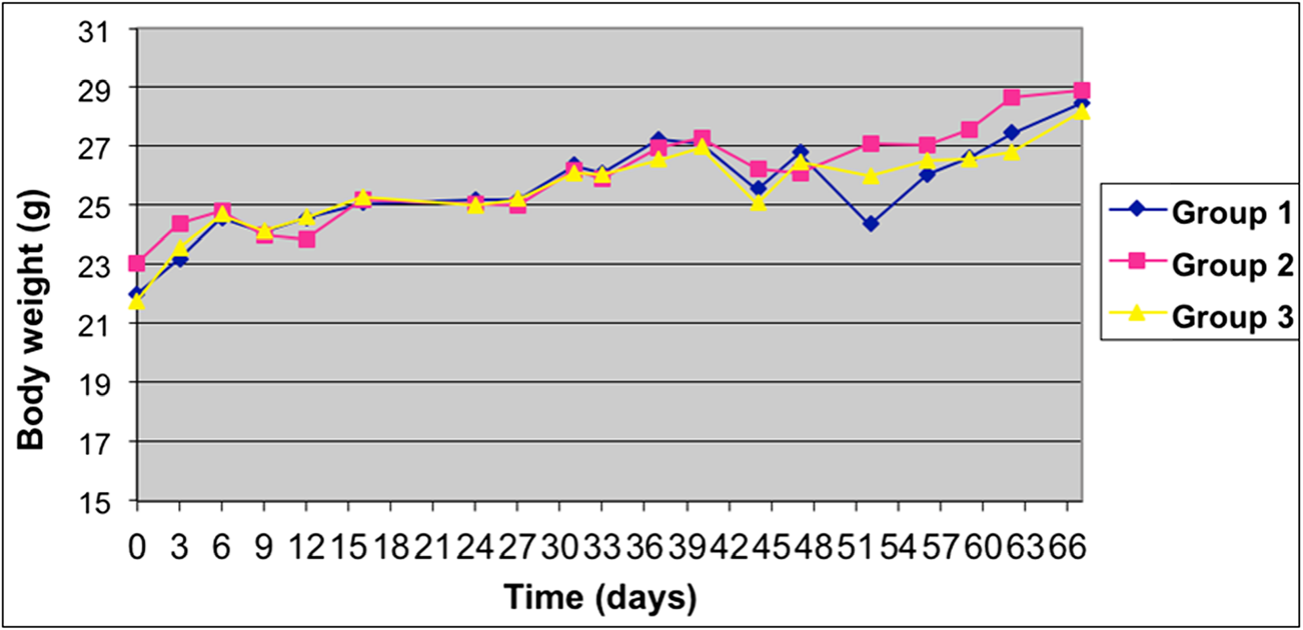
Mean weight of the mice after the inoculation of the cancer cells. Group 1: treatment by cisplatin (control); group 2: treatment with Re-diselenoether complex; group 3: treatment by Re-diselenoether complex + cisplatin

There was no sign of clinical toxicity in all groups according to the body weight of the mice, as depicted in Fig. 8. There was a death, but one in each group, between days 26 and 39 before the injection of cisplatin, that could be probably attributed to the pulmonary metastases (an autopsy was performed in 2 mice, revealing a great number of metastases). Thus, the dose of 10 mg/kg/24 h of Re-diselenoether appeared to be well-tolerated.

#### Biodistribution of Re-diselenoether complex in mice

The efficacy of the complex has been established in the animal experiment at a non-toxic dose of 10 mg/kg/d for 4 weeks, both on the primitive tumors and on the metastases. The biodistribution of Re and Se has already been published at this oral dose of 10 mg/kg/d versus 40 mg/kg/d of Re-diselenoether, and it was shown that the oral administration allows a good tissue uptake of Re and Se with a dose-effect [9]. Mice were treated with Re-diselenoether at the dose of 10 mg/kg (corresponding to 3.3 ppm in Re and 2.8 ppm in Se), and 40 mg/kg (13 ppm in Re and 11 ppm in Se), once-a-day for a period of 4 weeks. The distribution study revealed a Re concentration in the liver of 7.1 μmol/kg wet tissue (1.3 ppm) at the dose of 10 mg/kg and 19.8 μmol/kg wet tissue (3.4 ppm) at the dose of 40 mg/kg. Compared to the liver, lower concentrations were recorded in the kidney, namely 4.3 μmol/kg wet tissue (0.8 ppm) at the dose of 10 mg/kg and 8.8 μmol/kg wet tissue (1.6 ppm) at the dose of 40 mg/kg. Regarding Se, the following concentrations (corrected from the essential Se found in the tissues of untreated mice) were recorded : in the liver, 12.9 μmol/kg wet tissue (1.0 ppm) at the dose of 10 mg/kg, 31.1 μmol/kg wet tissue (2.5 ppm) at the dose of 40 mg/kg; in the kidney, 7.0 μmol/kg wet tissue (0.5 ppm) at the dose of 10 mg/kg and 8.8 μmol/kg wet tissue (0.7 ppm) at the dose of 40 mg/kg. These results deserve the following comments. A clear dose-effect of the drug was observed. Indeed, a 4-fold increase of the administered dose of Re-drug **1** (from 10 to 40 mg/kg/d) resulted in a 2.8-fold increase of the Re concentration in the liver and a 2.0-fold increase in the kidney. On the other hand, the Se/Re molar ratios in the liver were 1.8 at the dose of 10 mg/kg and 1.6 at the dose of 40 mg/kg. These ratios are quite close to the 2.0 Se/Re ratio found in the drug (two atoms of Se and one atom of Re per molecule). This observation suggests that the drug might be stored in the liver, more or less as it stands. However, in comparison with the liver, lower Se/Re ratios were recorded in the kidney (1.6 at the dose of 10 mg/kg and 1.0 at the dose of 40 mg/kg). These ratios revealed, as expected, a notable excretion/metabolization of the drug at the kidney level.

#### Mechanisms of action of diselenoether complex 1

As both Re and Se are well taken up by tissues, it is possible to consider that these two elements will contribute to the antitumor effects by the combination of their different mechanisms of action. The main biological effects of Re are the formation of adducts (single or double strands) with proteins or DNA. These interactions have been extensively studied by Alberto [20, 21, 26] and Zobi [27]. Re can bind to DNA adenine through the N1, N6 positions [28] or to guanine through the N7 position [29, 30], resulting in Re/nucleotide 1:1 or Re/nucleotide 1:2 adducts. Reaction of Re-diselonether **5** with 9-methylguanine used as a simple model of DNA bases clearly established its ability to produced Re/nucleotide 1:1 or Re/nucleotide 1:2 adducts. In contrast to cisplatin, binding of Re drugs to one or two bases is reversible, the Re-adducts having proved less stable than Pt-adducts. In fact, the formation of octahedral Re-adducts may be disfavored since they are generally more bulky and more sterically crowded than square-planar Pt-adducts. The possibility to administer the Re-diselenoether as a continuous oral administration offers an advantage upon cisplatin, which generally needs to be administered through a single injection.

Although the anti-carcinogenic properties of Se are now well established, the modes of action of this element are still a subject of discussion, since they are very complex and not fully understood. However, we can emphasize on the mechanisms of action of Se on redox potential status, inflammation, immunity and cell signaling pathways including the consequences on cell apoptosis, DNA repair and metal detoxification, angiogenesis, metastasis, and finally the effects on the tumor growth.

Among the mechanisms of action of Se, its effects on the oxidative system are perhaps the most important. Se is mainly an anti-oxidant, via the selenoproteins, such as glutathione peroxidase (GPx) and thioredoxine reductase (TrxR). In fact, the existence of a systemic pro-oxidant status in patients with breast cancer is well-established [31], depending on the stage of the disease [32] and on the molecular subtype [33]. A single systemic profile was found in patients with triple negative breast cancer with higher NO levels among subtypes [33]. An other antioxidant, the superoxide dismutase (SOD), has also been proposed to fight against cell proliferation [34]. Ovarian cancer patients resistant to treatments by carboplatin/paclitaxel have a lower level of antioxidant response activation compared to sensitive patients [35], and to restore the oxidative status could increase the efficacy of anticancer cytotoxic drugs. By contrast, the common cytotoxic agents are pro-oxidant drugs, like paclitaxel and doxorubicin [36]. In this respect, high concentrations of Se may produce reactive oxygen species (ROS) and lead to apoptotic cell death by inducing oxidation and cross-linking of protein thiol groups essential for cell survival [37]. According to Jamier et al. [38], Se-based agents could turn the oxidizing redox environment present in certain cancer cells into a lethal cocktail of reactive species, that push these cells over a critical redox threshold and ultimately kill them through apoptosis. This kind of toxicity is highly selective: healthy cells remaining largely unaffected, since changes to their naturally low levels of oxidizing species produce little effect. The balance between pro-oxidative and anti-oxidative effects of Se compounds is still unclear, but it is obvious that the redox potential of cancer cells needs to be taken into account to evaluate the treatments by Se compounds. A very interesting display thiol-proteomics approach to characterize global redox modification of proteins by Se has been proposed by Park et al. [39].

As a second mechanism of action, Se, mainly as selenoproteins, plays an important role in inflammation [40, 41]. There is a strong interaction between inflammation and cancer, and Pt-drugs have even been designed with the aim of targeting NF-kappa B signaling pathways [42]. Se compounds could have an impact on the inflammatory status through the inactivation of NF-kappa B [43, 44]. The interactions between cancer stem cells (CSC) and inflammation are also of a great importance [45] for the development of cancer and its resistance to therapeutic agents. Due to its effect on inflammation, we could expect a role of the Re-diselenoether complex in the growth and activity of CSC.

Studies with Se-compounds indicated that Se may also have positive effects on immune response [46–48], and more specifically on the activity on natural killer (NK) cells [49–51]. Methylselenol, which is the active metabolite of organic Se compounds, has already been shown to regulate the expression of NKG2D ligands by MDA-MB231 and MCF-7 cells [52]. These ligands are involved in the recognition of the malignant cells by NK cells [53–58]. Selenoproteins also mediate T cell immunity through an antioxidant mechanism [59]. Se plays also an important role as an anti-inflammatory agent by tightly regulating the expression of pro-inflammatory genes in immune cells [60].

The role of Se compounds on signaling pathways involved in the development of cancer has become a very attractive area of research. Se-compounds are thought to modulate several kinases. The PI3K/AKT pathway appears as a common target for Se-compounds, but they may modulate different kinases at the same time and their effectiveness depends on the genetic background of the tumor cells [61, 62]. All the Se-compounds did not exhibit kinase inhibitory activity. The type of kinase inhibition greatly depends on the Se derivative. The kinases modulated by S- and Se- derivatives include MAP, ERK, JNK, Akt, Cdc2, Cyclin B1 and Cdc25c amongst others [17]. Therefore, there is a great need for testing the Re-diselenoether complex on different tyrosine and serine/threonine kinases, especially in breast cancer cell lines.

Due to the molecular and biological effects of Se and selenoproteins, there is an expected benefit in cancer patients, not only on the primitive malignant tumor growth, but also on angiogenesis [63, 64] and metastasis [65]. However, the exact schedule of treatment needs to be clarified for each cancer disease, with the help of different markers that remain to be better identified. Plasma Se levels, which have already been investigated in a cohort of breast cancer patients [66], could be useful to monitor the therapy. Whatever the modes of action of these elements, one can argue that there existed a synergistic effect between Re and Se partners accounting for the remarkable, promising antitumor activity of Re(I)- diselenoether complex, already patented in Europe [67].

In summary, Re-diselenoether complex is a promising new metal-based anticancer drug for the treatment of patients with metastatic breast cancer. It proved to efficiently reduce tumor cell division in vitro at a low concentration of 10 μM. It may be orally administered, and the recommended dose is a non-toxic dose of 10 mg/kg/d for a treatment of at least 4 weeks. The efficacy may result from the activity of both Re and Se on key targets of the cancer cells and their microenvironment. Among the mechanisms of action, we confirmed the effects on DNA, due to the Re atom. The effects on the immune system, the redox status, the inflammation and cell signaling pathways attributed to the Se component will be investigated in further studies with models of hormone-independent (MDA-MB231) and hormone-sensitive (MCF-7) metastatic breast cancer.

## Electronic supplementary material

Supplementary Fig. 1 (DOCX 33 kb)

Supplementary Table 1 (DOCX 11 kb)
