# Supplementary material for: Antitumor activity of a rhenium (I)-diselenoether complex in experimental models of human breast cancer
Source: Invest New Drugs. 2015 Jun 26;33(4):848–60. doi: 10.1007/s10637-015-0265-z (PMC4491361; doi:10.1007/s10637-015-0265-z)
Supplement: Supplementary file 1 — (DOCX 33 kb) [file 10637_2015_265_MOESM1_ESM.docx]

Supp 1 : Structures of the observed ions in mass spectrum
