# Supplementary material for: Antitumor activity of a rhenium (I)-diselenoether complex in experimental models of human breast cancer
Source: Invest New Drugs. 2015 Jun 26;33(4):848–60. doi: 10.1007/s10637-015-0265-z (PMC4491361; doi:10.1007/s10637-015-0265-z)
Supplement: Supplementary file 2 — (DOCX 11 kb) [file 10637_2015_265_MOESM2_ESM.docx]

Table I: Evolution of the mice tumor volume, in mm^3^ (mean ± SEM) after IP administration of cisplatin (group 1), oral administration of Re (I)- diselenoether complex with the dose of 10 mg/kg/d (group 2), oral administration of Re (I)- diselenoether with the dose of 10 mg/kg/d + IP injection of cisplatin (group 3).

| Days | Group 1 | Group 2 | Group 3 |
| --- | --- | --- | --- |
| 0 | 0 ± 0 | 0 ± 0 | 0 ± 0 |
| 3 | 1 ± 2 | 0 ± 0 | 1 ± 2 |
| 6 | 1 ± 2 | 0 ± 0 | 1 ± 3 |
| 9 | 3 ± 3 | 3 ± 4 | 3 ± 5 |
| 12 | 5 ± 6 | 4 ± 4 | 3 ± 6 |
| 16 | 6 ± 9 | 4 ± 6 | 5 ± 8 |
| 24 | 26 ± 21 | 9 ± 13 | 11 ± 16 |
| 27 | 20 ± 27 | 12 ± 15 | 13 ± 18 |
| 31 | 28 ± 40 | 17 ± 24 | 17 ± 23 |
| 33 | 31 ± 40 | 19 ± 22 | 19 ± 28 |
| 37 | 41 ± 48 | 16 ± 20 | 21 ± 25 |
| 40 | 55 ± 56 | 16 ± 20 | 28 ± 33 |
| 44 | 62 ± 67 | 20 ± 27 | 35 ± 41 |
| 47 | 70 ± 108 | 7 ± 15 | 39 ± 63 |
| 52 | 102 ± 30 | 7 ± 2 | 101 ± 21 |
| 56 | 114 ± 32 | 6 ± 3 | 105 ± 21 |
| 59 | 114 ± 30 | 5 ± 2 | 127 ± 28 |
| 62 | 122 ± 34 | 5 ± 3 | 160 ± 34 |
| 67 | 138 ± 38 | 5 ± 3 | 183 ± 36 |
